# A Bio-Hybrid Tactile Sensor Incorporating Living Artificial Skin and an Impedance Sensing Array

**DOI:** 10.3390/s141223781

**Published:** 2014-12-10

**Authors:** David Cheneler, Elisa Buselli, Domenico Camboni, Carl Anthony, Liam Grover, Michael John Adams, Calogero Maria Oddo

**Affiliations:** 1 School of Engineering, Lancaster University, Lancaster LA1 4YW, UK; 2 The BioRobotics Institute, Scuola Superiore Sant'Anna, Polo Sant'Anna Valdera, Viale Rinaldo Piaggio 34, Pontedera 56025, PI, Italy; E-Mails: e.buselli@sssup.it (E.B.); d.camboni@sssup.it (D.Ca.); 3 School of Mechanical Engineering, Birmingham University, Birmingham B15 2TT, UK; E-Mail: c.j.anthony@bham.ac.uk; 4 School of Chemical Engineering, Birmingham University, Birmingham B15 2TT, UK; E-Mails: l.m.grover@bham.ac.uk (L.G.); m.j.adams@bham.ac.uk (M.J.A.)

**Keywords:** bio-hybrid sensors, bioimpedance, haptics, tactile sensors, artificial touch, artificial skin, microfluidics

## Abstract

The development of a bio-hybrid tactile sensor array that incorporates a skin analogue comprised of alginate encapsulated fibroblasts is described. The electrical properties are modulated by mechanical stress induced during contact, and changes are detected by a ten-channel dual-electrode impedance sensing array. By continuously monitoring the impedance of the sensor array at a fixed frequency, whilst normal and tangential loads are applied to the skin surface, transient mechanotransduction has been observed. The results demonstrate the effectiveness and feasibility of the preliminary prototype bio-hybrid tactile sensor.

## 1. Introduction

Current biomimetic artificial hands for robotic and neuro-prosthetic applications target the execution of human-like tasks such as grasping and manipulation. In order to achieve demanding tasks it is necessary to incorporate sensory systems mimicking the human sense of touch. This means that artificial hands should be equipped with both a sensing system capable of acquiring physical measurements (such as the contact force and soft tissue deformation map) which characterize the tactile stimulation through physical interaction, and the capability to translate the measured quantities into one or more touch-specific perceptual qualities such as pressure, shape, roughness, softness and curvature [1].

The tactile transduction of human hands is complex, involving populations of mechanosensitive receptors of the distal finger pad and the remainder of the skin throughout the different layers [2–5]. Mechanoneurotransduction occurs when an external stimulus transfers energy to the human finger pad during contact via mechanical stress, which elicits sequences of electrical discharges that are transmitted to the brain via the neural afferent pathways and code the stimulus in a form that can be perceived [6].

Both synthetic and bio-artificial skins have been explored as artificial touch approaches and a large number of transduction mechanisms have been reported in the literature [1]. Microfabrication technologies allow the design and fabrication of novel miniaturized sensors made from sophisticated materials and with integrated processing capabilities. Biological Micro-Electro Mechanical Systems (BioMEMS) and related devices, which utilize artificial skin, can be fabricated with different classes of inorganic and bio-engineered (both macromolecules and tissues) materials, such as microelectronics-related materials (silicon and glass), plastics and other polymeric materials (e.g., poly(dimethylsiloxane) elastomers) and biological materials such as proteins, cells and tissues [7–9]. Biological and other soft materials for skin-like surfaces with integrated sensors are desired in biomimetic artificial hand design because a soft skin can protect embedded sensors, increase the size of the contact area and provide more effective tactile information, and increase the contact friction coefficient, thus ensuring higher stability whilst grasping.

Bio-hybrid tactile sensors may be classified as all those for which the transduction mechanism is dependent on the conversion of mechanical stress by the biological component into a form that is more readily detected by pertinent electronics. In this way, the mechanotransduction processes in the biological component can be exploited without having to employ complex interfaces. This definition can be compared to fully synthetic skin based tactile sensors; in that case the ‘skin’ has the function of packaging and does not play a major active role in the transduction mechanism, being mainly involved in the transmission of the mechanical stimuli from the environment to the sensitive area. Furthermore, fully biological tactile sensors should use the mechanoelectrotransduction mechanisms inherent in mechanoreceptor cells in order to sense contact.

Bio-hybrid and fully-biological tactile sensing systems can further enhance the level of bio-mimicry in artificial hands: the use of skin analogues could enable a prosthetic hand to be biologically similar to the natural finger and therefore more likely to be accepted by a human subject. In addition, fully-biological approaches for tactile sensing systems in neuro-prosthetics could permit straightforward neuromorphic coding [10,11] of tactile information, that may be obtained directly by a neuro-inspired spike-based architecture, which could be derived from living mechanoreceptors and would enable direct connection of the artificial tactile system to the central nervous system. Finally, bio-hybrid and fully-biological tactile systems could show self-healing properties, which would overcome some intrinsic limitations of synthetic approaches, in case that microfluidic vascular-like structures can be introduced in the construct [12].

Table 1 summarizes the possible approaches that can be used to fabricate a tactile sensing system: they include synthetic and bio-artificial approaches (classified as bio-hybrid and fully-biological). Advances in the development of synthetic tactile sensing systems have been presented by several research groups, while bio-hybrid and biological artificial tactile sensors are a more recent and less established paradigm. For a comprehensive review of state of the art on artificial tactile sensing refer to [13], in which classification and properties of available solutions are described.

In the current paper, we describe the development of a bio-hybrid tactile sensor array. The system incorporates a skin analogue comprised of alginate (a hydrogel commonly used as a cell encapsulation matrix [14,15]) encapsulated fibroblasts, which are known to be mechanosensitive [16–18]. It is well known that connective cells are an integral part of extracellular matrix production and regeneration [19–21]. Indeed, they are the main biological component in fundamental healing processes such as fibrosis and collagen production. If this function can be achieved *in vitro* conditions, in a system such as the one presented in this paper, then a self-healing sensor could be implemented by integrating the required system for homeostasis. This issue is under study by several research groups [16,22–25] and the implications are certainly worth investigating.

In the system presented in this study, the electrical properties of the skin analogue are affected by mechanical stress induced during contact, and this change in electrical properties is detected by a ten-channel dual-electrode impedance sensing array. The sensors are supported by a microfluidic system designed to maintain the viability of the encapsulated cells. By continuously monitoring the impedance of the sensor array at fixed frequency whilst normal and tangential loads are applied to the skin surface, transient mechanotransduction has been observed. In this manner, the feasibility of the bio-hybrid tactile sensor has been demonstrated.

## 2. Bio-Hybrid Tactile Sensor Array and Integration within a Dynamic Tactile Stimulation Platform

### 2.1. Bio-Hybrid Tactile Sensor Array

#### 2.1.1. Overview of Bio-Hybrid Tactile Sensor Array

**Figure 1.**
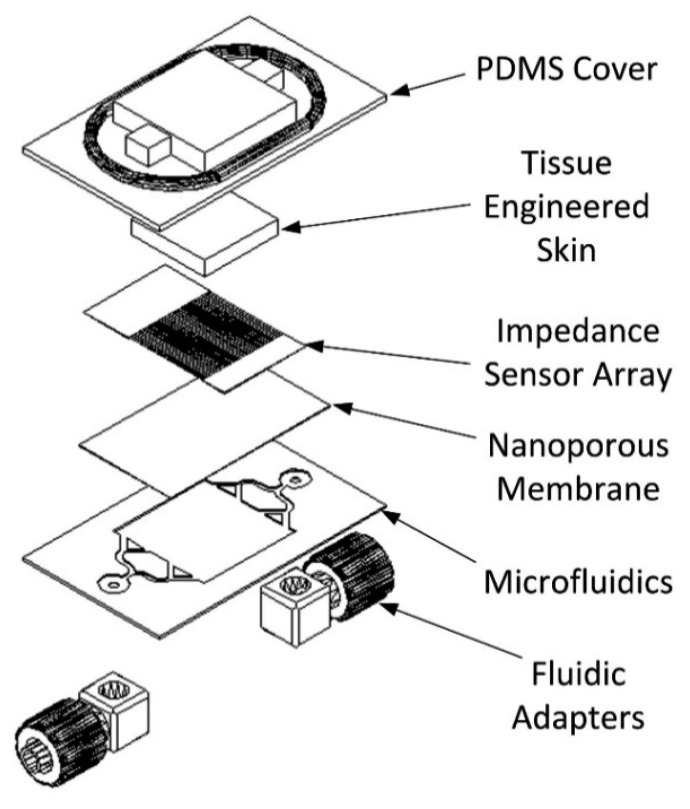
Exploded schematic of the bio-hybrid tactile sensor array.

The developed bio-hybrid system is comprised of multiple layers. An exploded view of the construct is shown in Figure 1.

**Figure 2.**
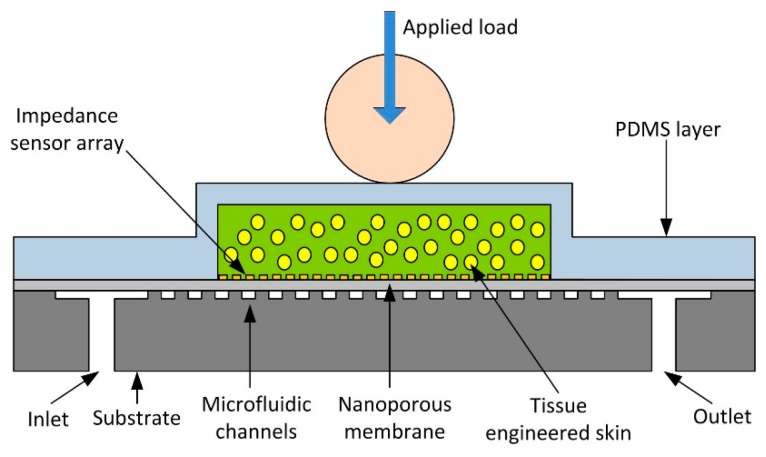
Cross-section of the bio-hybrid tactile sensor array. Not to scale.

The device consists of a system of microfluidic microchannels etched into a silicon substrate designed to supply the skin analogue with nutrients and maintain its long-term viability. The channels lead into a chamber with a regular array of 50 μm diameter micropillars that allow flow whilst supporting a polycarbonate nanoporous membrane. The membrane is 30 μm thick with an irregular array of 200 nm diameter through-thickness holes etched. On the membrane is an impedance sensor array consisting of ten parallel electrodes, each pair forming an impedance sensor, shielded with ground lines. The membrane with electrodes forms a layer upon on which the skin analogue is supported while allowing nutrient media to diffuse through to the cells. The cells were cultured and encapsulated in alginate, a hydrogel with mechanical properties comparable to the dermal layer of skin. The microfluidic channels are sealed and the cells are confined to a well using a poly(dimethylsiloxane) (PDMS) cover plate. The PDMS effectively becomes the epidermis layer providing a robust surface to conduct experiments whilst transmitting stresses and protecting the encapsulated skin analogue. The PDMS also serves as a seal containing any excess fluid. A cross-section of the assembled system is shown in Figure 2.

Human skin is far more complex than the tissue used in this work, but the mechanical properties are comparable: for instance the Young's modulus for alginate hydrogel prepared in the same way to that used in this paper is *c.a.* 200 kPa [26] and the Young's modulus for the 3 mm thick PDMS is *c.a.* 600 kPa with a Poisson's ratio tending to 0.5, *i.e.*, practically incompressible, being an elastomer [27], while the dermis has a Young's modulus of 420–850 kPa [28]. Alginates are commonly used as dermal analogues due to their comparable properties and ease of use as an extracellular matrix to encapsulate cells and so are pertinent as a model material [29]. Due to the finite time required for the solvent to flow through the polymer network under compression, the tissue behaves approximately like an incompressible elastic solid at very short times under an instantaneous application of force. Detailed modelling of hydrogels is non-trivial, particularly when contact is involved, with appropriate nonlinear multiphasic constitutive models still under development, and is outside the scope of the feasibility study presented in this paper.

#### 2.1.2. Microfluidics and Impedance Sensor Array

The microfluidic system was fabricated in several components that were then assembled. The microfluidic base was fabricated in silicon using deep reactive ion etching. The fabrication process was as follows:

- Evaporate primer (Chestech, Rugby, UK) onto 525 μm thick silicon wafer (Si-Mat, Kaufering, Germany) for 120 s.
- Spin on 9 μm thick layer of SPR220-7 photoresist (Chestech) onto wafer using the following speed cycle:
  500 rpm for 15 s, ramp 10 rpm/s.
  2000 rpm for 30 s, ramp 50 rpm/s.
  500 rpm for 5 s, ramp 50 rpm/s.
- Softbake wafer on hotplate for 90 s at 95 °C.
- Expose for 45 s under UV in the PLA-501FA Mask Aligner (Canon, Melville, NY, USA) to define the inlet and outlet holes.
- Develop in MF26A (Chestech) for ∼60 s.
- Rinse with DI water.
- Blow dry with nitrogen.
- Cover a 525 μm silicon handle wafer with Revalpha thermal release tape (Nitto Denko, Osaka, Japan) and trim to size.
- Align patterned wafer with handle wafer and attach to thermal tape.
- Descum the wafer to remove residual photoresist using oxygen plasma in the Multiplex ICP DRIE etcher (Surface Technology Systems, Newport, UK). O_2_ rate = 100 sccm, 13.56 MHz platen power = 800 W, RF coil power = 600 W, duration = 30 s.
- Etch through the wafer using DRIE etcher and the Bosch process for 175 min using the parameters given in Table 2 (etch rate ∼3 μm/min).
- Remove photoresist using oxygen plasma. O_2_ rate = 100 sccm, 13.56 MHz platen power = 800 W, RF coil power = 600 W, duration = 5 min.
- Repeat same UV lithography process as before with second mask to define microfluidic channels and micropillar array.
- Repeat DRIE process as before but for 100 min to a depth of 300 μm.
- Remove photoresist using oxygen plasma. O_2_ rate = 100 sccm, 13.56 MHz platen power = 800 W, RF coil power = 600 W, duration = 10 min.
- Heat handle wafer to 120 °C on hot plate to release thermal release tape and remove handle wafer.

**Figure 3.**
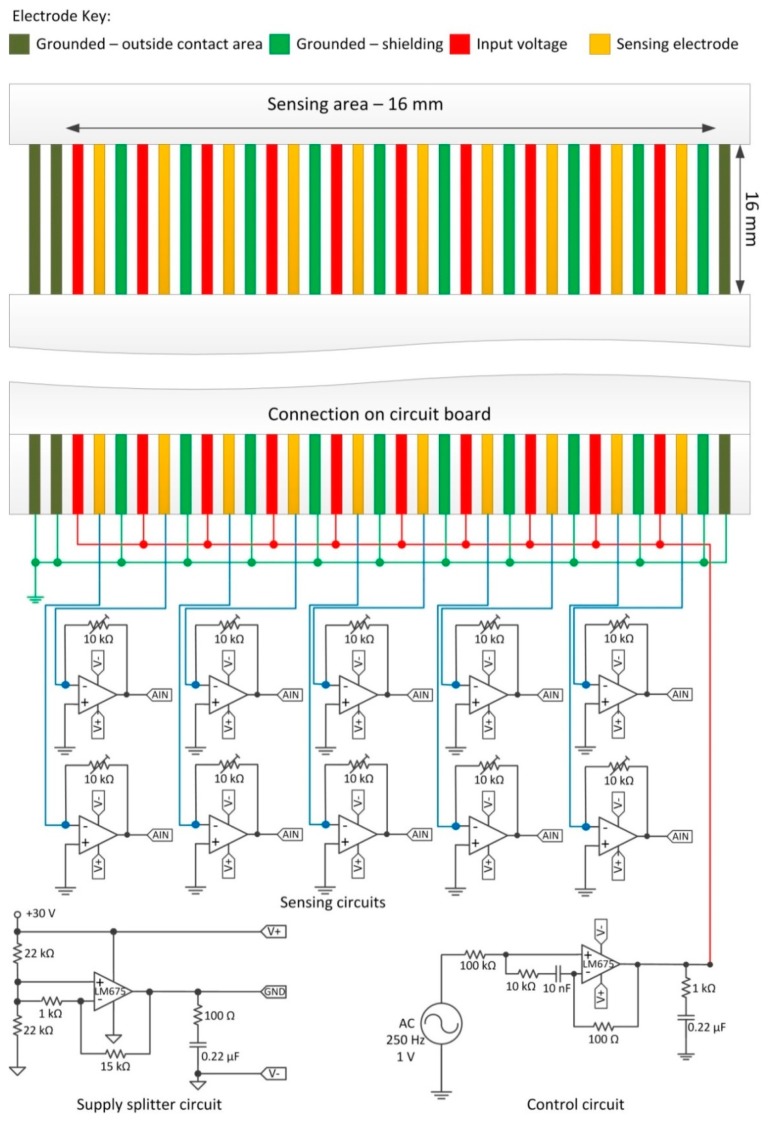
The monitoring circuitry for the impedance sensor array.

The impedance sensor array is a ten channel linear configuration of electrode pairs interspersed with ground lines to minimise electrical cross talk between the sensors. The electrodes consisted of tin plated copper conductors insulated between polyester based tape (Pro Power, Farnell, Leeds, UK). Each electrode was 0.3 mm wide with a pitch of 0.5 mm. The array was fabricated by means of a chemical etch process applied to a 210 mm flat flexible cable (FFC): a section of the FFC insulation layers was etched using a methyl trichloride/phenol solution at a ratio of 2:3 by weight at 110 °C. This was done to expose a section of the wires, nominally 16 mm long. This section was placed onto the nanoporous membrane in direct contact with the skin analogue. The end of the FFC was connected to the interface circuitry as shown in Figure 3: one electrode of the sensing pair was connected to the input signal via a high power buffer amplifier to ensure the voltage remains constant should the resistance between the electrodes drop significantly.

The input voltage was maintained as an AC signal with a frequency of 250 Hz and amplitude of 1 V. The other electrode was connected to the negative terminal of a transimpedance amplifier. This converts the current travelling between the electrode pairs into a voltage with a gain factor proportional (with a negated sign) to the resistance value in the transimpedance circuit, in this case 10 kΩ. The output of all the transimpedance amplifiers were streamed directly into a DAQ.

In this work, impedance monitoring was used as this method is potentially applicable with integrated electronics in an embedded sensing system. Impedance measurements are nowadays a standard to evaluate cellular response during culture. Traditional protocols are cells counting by microscopic observation, quantifying cellular components, live/dead fluorescent dye staining, and analysis of metabolites synthesized by the cultured cells. These methods are labour-intensive and time-consuming and in same case (e.g., for fluorescent dye) can damage the cells. On the contrary, impedance measurement is a non-invasive method and the outcomes are electrical signals; usually, a pair of electrodes is utilized to measure the impedance change caused by the biological substances and the output signals are easily interfaced with external devices [30].

#### 2.1.3. PDMS Cover Layer

**Figure 4.**
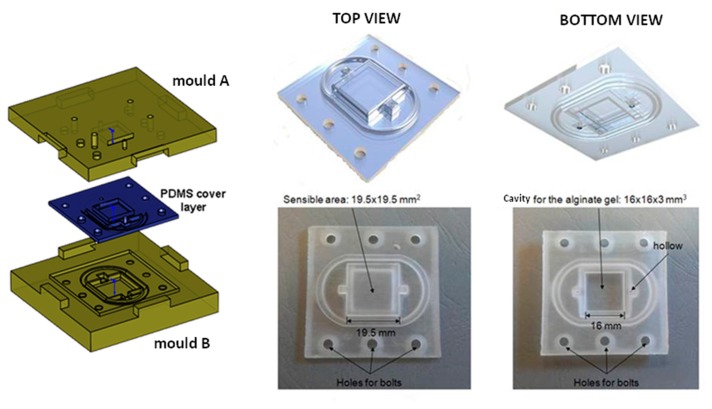
The PDMS cover layer: Fabrication procedure and final prototype.

The PDMS cover layer was designed to house the skin analogue, forming a watertight seal around the entire microfluidic system. PDMS is elastic, soft and gas permeable, thus allowing the respiration of cells. In addition to being a basic seal, it is also tough and durable and therefore it had a role similar to the epidermis layer on human skin. It was fabricated by mixing a two-part solution in a 10:1 ratio (Sylgard 184, Dow Corning, Midland, MI, USA) and pouring it into a mould fabricated using a 3D printer. The mould was compressed and the PDMS was polymerized in an oven at 80 °C for 4 h (see Figure 4).

### 2.2. Protocol for Production of Alginates

**Figure 5.**
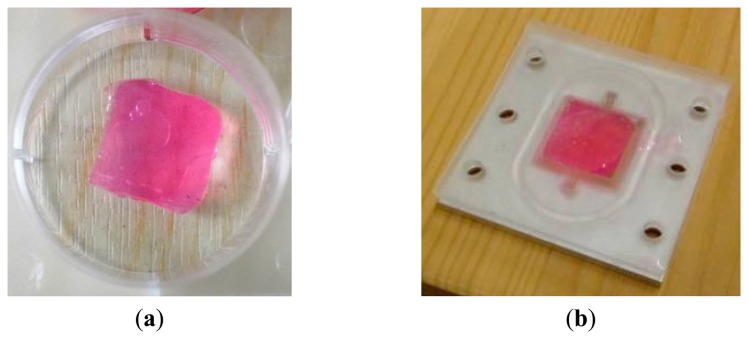
(**a**) Alginate hydrogel sample; (**b**) sample incorporated into the well of the PDMS cover layer. The pink colour is given by the culture medium, which contains Phenol Red, a pH indicator used to monitor the tissue during the encapsulation of the fibroblasts. It was included in the acellular tissues as well to ensure the composition was as comparable to the cellular tissues as possible.

All chemicals were sourced from Sigma Aldrich (St. Louis, MO, USA). 5% w/v alginate solutions were prepared by mixing sodium alginate with distilled water at the appropriate amounts at a temperature of 75 °C for 2 h. A 2 mol/L CaCl_2_ solution was also prepared using distilled water. Both solutions were autoclaved for 15 min at a temperature of 120 °C in order to be sterilized for the cell encapsulation procedure. Gelation filter paper discs were saturated with the CaCl_2_ solution and placed at the bottom of a 12-well plate. Alginate was added to form a 3 mm thick layer before another saturated filter paper disc was applied to the upper surface of the alginate solution. The CaCl_2_ solution diffused into the alginate causing it to cross-link ionically. After gelation the alginate was removed from the well, the filter discs discarded and the alginate diced into 16 × 16 mm samples and incorporated into the PDMS well (see Figure 5).

### 2.3. Protocol for Encapsulation of Cells in Alginate

MC-3T3 fibroblasts were cultured for a week in supplemented Dulbecco's Modified Eagles' Media (DMEM) which consisted of: high glucose (4.5 g/L) DMEM media, 10% foetal bovine serum, 4 mM L-glutamine, 22 mM HEPES buffer, and 1% pen-strep solution and fed every second day of the cell culture. Cells were cultured in T-flasks at 37 °C with 5% CO_2_, in a humidified incubator. The fibroblasts were trypsinised on day 7 of the cell culture from the T-flasks with Tryple select (Life Tech, Carlsbad, CA, USA) in order to be encapsulated in the alginate hydrogels. For the cell encapsulation procedure, a cell density of 10^6^ cells/mL was used since previous tissue engineering [29] and mechanotransduction [16] research showed that such a density encapsulated in 5% w/v alginate solutions effectively simulates the extracellular matrix of soft tissue allowing for nutrients, waste products and signalling molecules to effectively perfuse the scaffold and thus maintain viability of encapsulated fibroblasts. The cells were added and randomly dispersed throughout the alginate solution, prior to the alginate gelation step described in Section 2.2 and integration into the PDMS well. The approach used allows for cells to remain as discrete entities within the scaffold so that cell-cell agglomerations are avoided. This resulted in the viable cell number remaining constant throughout the testing period.

Fibroblasts are mechanosensitive and contain stretch sensitive cation channels that might participate in mechanical to electrochemical signal transduction such as changes in intracellular calcium concentration [31,32]. It was hypothesised that the inclusion of these cells would enhance the impedance response of the sensor due to this extra efflux of calcium ions upon the application of stress.

### 2.4. Integration within the Dynamic Tactile Stimulation Platform

**Figure 6.**
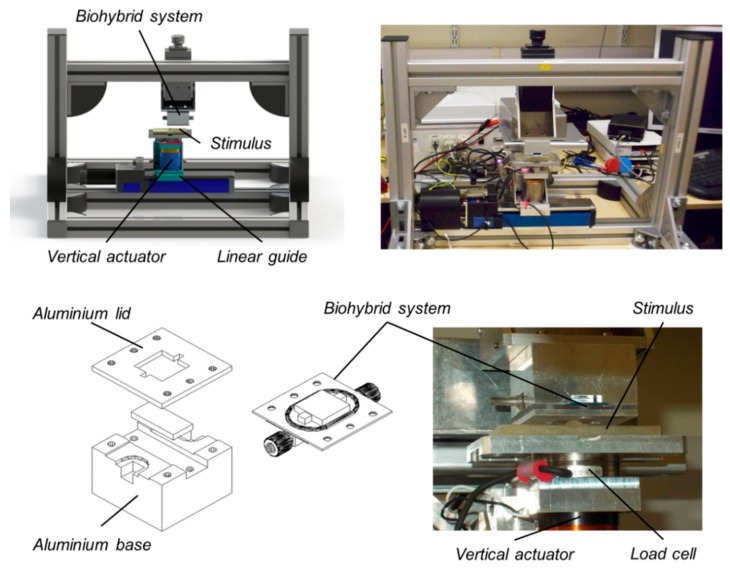
Integration of the bio-hybrid sensor in the dynamic tactile stimulation platform: Concept design and experimental set-up.

The dynamic platform (Figure 6) is a two DoF Cartesian manipulator, designed to indent and slide stimuli over the bio-hybrid tactile sensor. It is a tactile stimulation platform that has also been applied to artificial and human touch studies (via electrophysiological and psychophysical methods) [33,34].

The platform control architecture is multi-layered and hierarchical, partitioning the tasks between a general purpose PC, running a Graphical User Interface (Labview, National Instruments, Austin, TX, USA), and an embedded hardware-programmable logics (EP2C35 FPGA, Altera, San Jose, CA, USA) with custom hardware logic modules and a soft-core processor (Nios II/f, Altera) running C/C++ control and communication routines to implement the experimental protocols. The contact along the indentation axis is controllable in both position and force modes, while the motion along the sliding direction is under position/velocity control. The indentation is operated via a voice-coil actuator (NCC05-18-060-2X, H2W Tech. Santa Clarita, CA, USA), with a 12.7 mm stroke. The sliding motion is obtained via a linear guide (LTP 60.180.0804-02, SKF Multitec, Göteborg, Sweden) driven by a DC motor (RE35, Maxon Motors, Sachseln, Switzerland). A 0.1 μm position sensing resolution is obtained along the indentation axis (TONiC RELM T1011-15A, Renishaw, Wotton-under-Edge, UK), and 0.98 μm (MR Type L-1024, Maxon) along the sliding direction, while a 2.1 mN RMS noise is achieved for force sensing (Nano43, ATI Industrial Automation, Apex, NC, USA).

In order to test the bio-hybrid system, a custom aluminium element was designed to connect the sensor to the top of the dynamic platform. In this way, the bio-hybrid system takes the place of the finger in the human touch studies mentioned earlier. The stimuli were attached to the lower part of the dynamic platform, above the actuator, in order to be moved vertically along the indentation axis and translated horizontally along the tangential direction. The experimental procedure was designed so that the bio-hybrid sensor received stimulation profiles comparable to those that are applied to the human finger during ongoing electrophysiological and psychophysical passive-touch studies. During such experiments the sensor was interfaced to the readout electronics (transimpedance amplifier, described in Section 2.1.2) in order to record impedance data.

**Figure 7.**
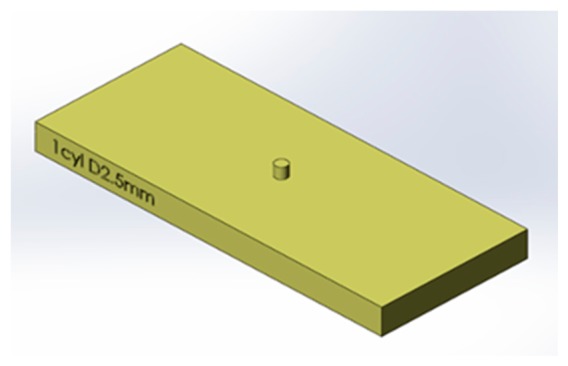
CAD drawing of the stimulus used in the tactile experiments.

During experimentation, normal and tangential loads were applied to the bio-hybrid system. This was achieved by sliding tactile stimuli across the surface of the bio-hybrid system using the dynamic tactile stimulation platform as shown in Figure 6. The stimulus consisted of a cylinder 2.5 mm in diameter and 2.5 mm in height fabricated using 3D printing (ProJet HD 3000, 3D Systems, Rock Hill, SC, USA) and fixed to an aluminium support. The geometry used in the investigation is shown in Figure 7.

## 3. Methods

### 3.1. Experimental Protocol

**Figure 8.**
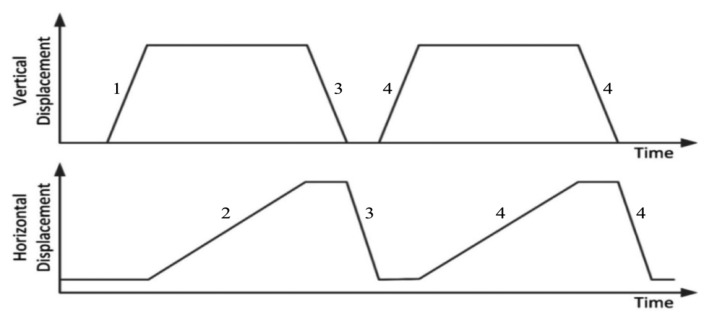
Schematic plot of the sliding protocol, showing normal and tangential motion of the stimuli: (1) move stimulus vertically into the sensor at 0.5 mm/s for 2 s (1 mm indentation); (2) slide stimulus across sensing area at 0.5 mm/s for 16 s (8 mm sliding); (3) retract at same speed and return to start location; (4) immediately repeat test.

The experimental protocol consisted in sliding experiments, inspired to psychophysical protocols reported in the literature [35]. The sliding protocol (Figure 8) consisted in an initial normal indentation and a tangential sliding motion. The large indentation depth was to ensure a significant change in impedance in this preliminary experimental evaluation, whereas the slow ramp time was to ensure the transient change in impedance was observable (phase 1 in Figure 8). The stimulus was displaced at constant velocity across the active sensing area (phase 2 in Figure 8). Then the stimulus was retracted and repositioned (phase 3 in Figure 8) and the experiment repeated (phase 4 in Figure 8).

### 3.2. Data Collection

**Figure 9.**
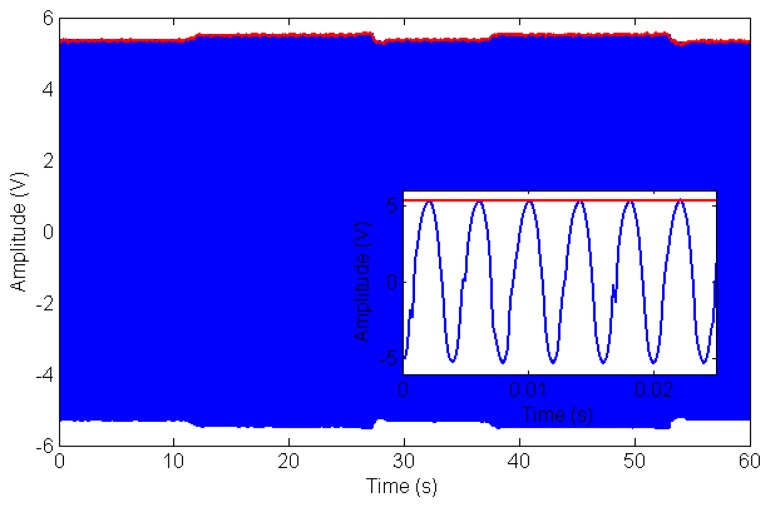
Typical raw data (blue line) from a single channel during experiments involving mechanical probing of the sensor while acquiring electrical data as detailed in Section 3.2. Data was taken from channel six during the first cellular test (see Figure 11). The red line indicates the amplitude of the 250 Hz carrier wave as a function of time calculated using the short time Fourier Transform. Modulation in amplitude of the carrier (reflecting changes of current flowing through the sensor and therefore impedance modulation) can be appreciated as a mechanical load is applied in the 10 s–30 s and in the 35 s–55 s time intervals. Inset: close-up of data showing the 250 Hz carrier.

The bio-hybrid sensor was composed by an array of ten electrode pairs with intermediate ground lines. One electrode was connected to a buffered AC power supply providing a signal of 1 V at 250 Hz (the control circuit in Figure 3). The other was connected to the transimpedance amplifier which acted as a virtual ground and converted the current passing between the electrodes into a voltage (see Section 2.1.2 for further details). The voltage from each channel transimpedance amplifer was streamed simultaneously into ten analogue inputs on a U6 DAQ (LabJack, Lakewood, CO, USA) at 5000 Hz. An example of the raw data for one channel during a typical experiment is shown in Figure 9.

The red line indicates the amplitude as calculated using FFT to extract the amplitude modulation over the 250 Hz sinusoidal driving signal (see Figure 3 for the conditioning circuitry). A window size of 0.1 s and a transform length of 4096 points was used in the Fast Fourier Transform algorithm [36]. Given a sampling frequency of 5000 Hz and a drive frequency of 250 Hz, this means the impedance data was averaged over 25 time periods with 20 samples per period. Considering the input-output characteristics (−10 kΩ gain) of the transimpedance amplifier, 1 V output voltage amplitude corresponded to −0.1 mA input current.

## 4. Data Elaboration

**Figure 10.**
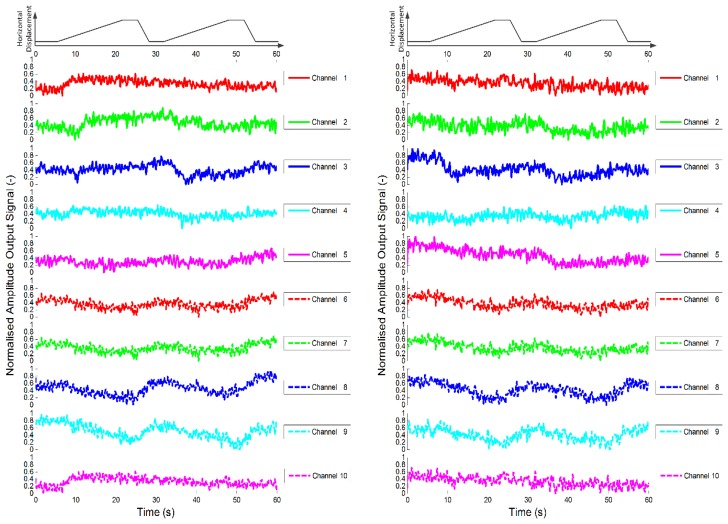
Plots of dynamic variation of peak value of short time Fourier analysis of sensor outputs during sliding test for two control sensors (*i.e.*, alginate only). The first rows of each plot show the horizontal displacement of the stimulus, which was a cylinder with 2.5 mm diameter.

As a control study, the sensor was first loaded with a cellular tissue, *i.e.*, pure alginate gel. Two data sets are shown in Figure 10 that demonstrate the response of each sensor channel as the stimuli is slid across the sensor surface twice in a manner described in Section 3.1.

**Figure 11.**
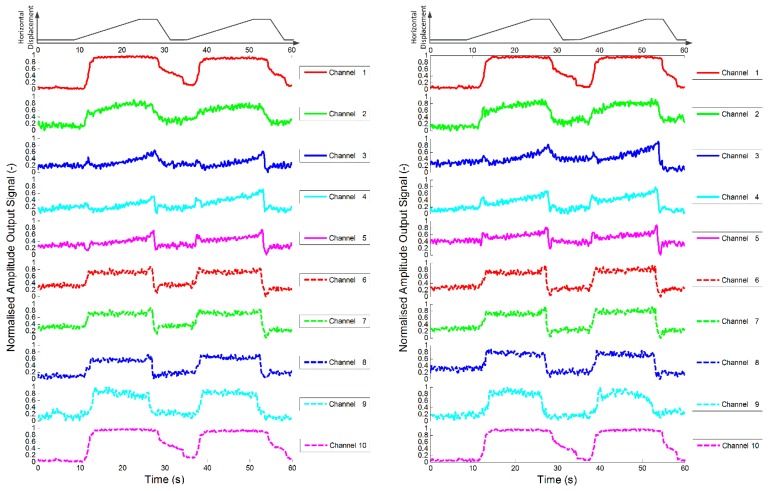
Plot of dynamic variation of peak value of short time Fourier analysis of sensor outputs during sliding test for two test sensors (*i.e.*, alginate with fibroblasts). The first rows of each plot show the horizontal displacement of the stimulus, which was a cylinder with 2.5 mm diameter.

As explained in the previous section, the raw data from each channel were analysed after each experiment using short time Fourier analysis and the amplitude of the output voltage for each channel is presented. Note that the pure alginate tissues are shown to alter the response of the sensor whilst under the influence of a force. The experiments were repeated, but this time with an alginate encapsulated fibroblast population, *i.e.*, a cellular tissue, incorporated into the test sensor. The results shown in Figure 11 demonstrate that the inclusion of fibroblasts significantly increases the change in the response of the sensors, with the indentation and sliding phases clearly visible in the data. It is also of note that the change in response appears to be quite consistent during repeated experiments.

In order to ascertain how consistent the response was for repeated experiments, a coefficient of determination was used to indicate how data sets compare [37]. The value of the coefficient of determination (*R*^2^) illustrates how different two data sets are. If they are identical, *i.e.*, the responses of the channels during two separate experiments are equal, *R*^2^ will have a value of 1. If the response of the channels are completely different during the two experiments the value will approach 0. The data given in Figures 10 and 11 are used as an example: each graph shows the response of each channel as the stimulus is slid across the sensor surface twice. Here, this is to be counted as two separate experiments so that Figures 10 and 11 represent eight experiments. For the analysis, a section of the data for each channel from two experiments is compared. This is achieved by ensuring that the two data sets are aligned such that the start of the stimuli occurs at the same time in both data sets and that the sets are of the same duration.

The data in the first data set are denoted as *y_i_* and the data from the second set are denoted as *f_i_*. The mean of the first data set is given as:

(1) $$\bar{y}=\frac{1}{n}\overset{n}{\underset{i=1}{\text{∑}}}y_{i}$$

where *n* is the number of data points, the most general definition of the coefficient of determination is:

(2) $$R^{2}\equiv 1-\frac{\overset{n}{\underset{i=1}{\text{∑}}}{\left(y_{i}-f_{i}\right)}^{2}}{\overset{n}{\underset{i=1}{\text{∑}}}{\left(y_{i}-\bar{y}\right)}^{2}}$$

Table 3 gives the coefficient of determination for each channel when comparing the response of the sensor incorporating just alginate gel using data from Figure 10 during the two passes for the first run (Test A), the two passes for the second run (Test B) and the first pass between separate runs (Test C). Note the coefficient of determination for the pure alginate gel (control sensor) is quite low due to the poor signal to noise ratio.

Table 4 shows the coefficient of determination (*R^2^*) for each channel of the test sensor (alginate encapsulated fibroblasts) given in Figure 11. Test D corresponds to the comparison of the response of the sensors during the two passes during the first run. Test E corresponds to the comparison of the response of the sensors during the two passes during the second run. Test F corresponds to the comparison of the response of the sensors during the first pass of the two runs. The coefficient of determination is much greater for the sensor with incorporated fibroblasts (test sensor) than in the device with only alginate (control sensor) suggesting a higher repeatability of results.

## 5. Additional Test Session

**Figure 12.**
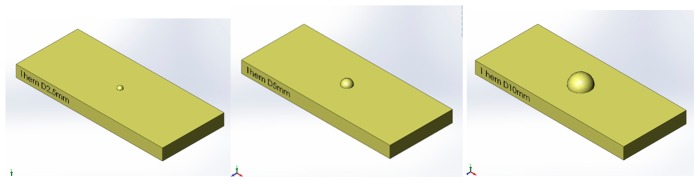
CAD design of the three hemisphere stimuli: 2.5 mm (**Left**); 5 mm (**Centre**) and 10 mm (**Right**) in diameter.

Finally, the same protocol was applied to stimuli of different shape and dimension. The new stimuli are three different diameter hemispheres; the spherical shape should avoid any eventual border effect. Figure 12 shows the three stimuli. We underline that, in the case of hemisphere, the height is different among the stimuli because it depends on the radius.

**Figure 13.**
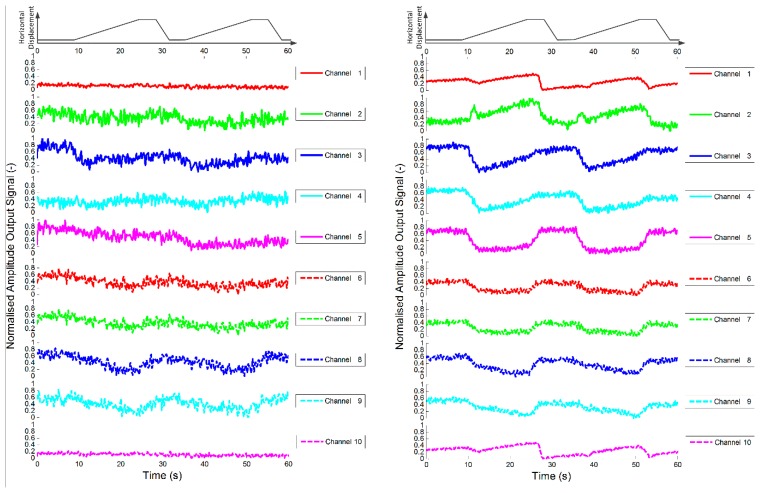

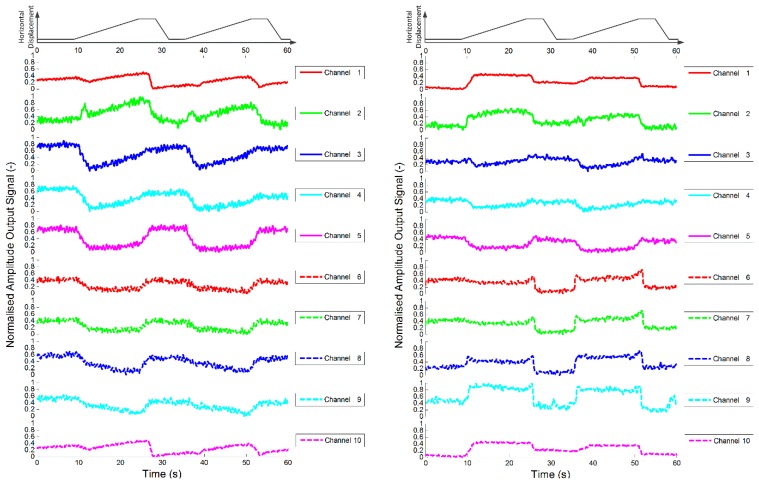
Plots of dynamic variation of peak value of short time Fourier analysis of test sensor (*i.e.*, alginate with fibroblasts) outputs during sliding test. The first rows of each plot show the horizontal displacement of the stimuli, that were a hemisphere 2.5 mm in diameter (top left), a hemisphere 5 mm in diameter (top right and bottom left), a hemisphere 10 mm in diameter (bottom right).

The tests were repeated for all the new stimuli as in the previous session, with the same experimental procedure and equipment. The results are shown in Figure 13.

It is worthwhile to observe that the 2.5 mm hemisphere has a height of 1.25 mm, and this is probably the reason of the very low response. For the other stimuli (2.5 and 5 mm in height) the response is consistent with the previous test session.

## 6. Discussion and Conclusions

The aim of this study is to build a bio-hybrid system and to contribute to open a novel and still immature research strands in the literature of tactile sensors, in which biological components are embedded in the synthetic sensor. Of course hybrid solutions, integrating biological components, present challenges with respect to long term viability and stability that are not posed by synthetic devices. The skin analogue (alginate + fibroblasts) presented here should stay in a controlled environment (temp. 37 °C, 5% CO_2_, humidity 90%) or it will decay in few hours. However, it has been shown that in controlled environments the tissue can remain viable for at least 150 days [29].

The fibroblast cells as used are known to be mechanosensitive and hence are supposed to have a role in the mechanotransduction process, as confirmed by experimental data. One mechanism by which the transduction could occur is via the opening of stretch-activated cation channels [38] that are associated with adhesion contacts. As a matter of fact, in previous studies mechanical stimulation has shown calcium influx and calcium-mediated intracellular signalling in fibroblasts [31,39,40]. Thus, in the present work, it was hypothesized that the inclusion of fibroblasts would enhance the impedance response of the sensor due to this extra efflux of calcium ions upon the application of stress.

Nevertheless, the control alginate sensor without fibroblasts also exhibited mechanotransduction (albeit with a lower degree of repeatability in comparison to the test sensor). This is because the alginate is essentially a polyelectrolyte gel with mobile ions resulting from not just the gelation process, but also from the presence of the inorganic salts in the DMEM [16]; these mobile ions result in the extracellular matrix being conductive. The deformation of the PDMS cover layer causes a change in the local polymer density and the global geometry of the tissue; this changes the possible conduction paths through the tissue and local diffusivity of the ions, hence the change in resistance [16,41,42]. Moreover, alginate is a polarisable molecule owing to the dissociation of the carboxyl group present in each monomer [43,44]. The resulting charge is what allows the gelation process to occur in the presence of divalent ions such as Ca^2+^. The charge means that the polymer chain is asymmetrical and also results in counterion condensation. The dissociation depends on the local electrochemical environment that in turn is affected by the mechanical stress on the alginate [44]. Therefore, in the acellular control sensor the force transduction mechanism will be a subtle interplay of all these processes, present within biological tissues as well.

BioTac [45–48] is one of the most advanced commercially available tactile sensors and it is interesting to qualitatively compare its characteristics with the current device since they both involve the measurement of impedance. It utilizes electrode pairs to measure the impedance of a weakly conducting, incompressible, low viscosity fluid (1 mol/L NaBr solution with diluted polyethylene glycol as a solvent) encapsulated in an elastomer case. Contact forces deform the elastomer case changing the possible conduction paths in the fluid which manifests as a change in resistance and hence impedance. The BioTac device also uses a pressure sensor to detect vibrations in the fluid. Similarly, in the current device, deformation of the PDMS layer results in an equivalent change in impedance. This partly arises from the alginate being a polyelectrolyte gel that behaves like an extracellular matrix. However, the current system has a biological component that is responsible for an additional change in impedance due to the activation of the ion channels in the cell membranes [16,17].

Part of the aim of this study is to use a simple model system in order to facilitate the study of mechanoreception with the view of building up complexity and understanding. The other purpose is to investigate the feasibility of developing a biomimetic force transduction mechanism as an alternative haptic sensor technology. For both purposes, the materials and assemblies used are acceptable. In any case, additional future efforts will focus on the development of a more accurate interface.

Qualitatively, the experimental results were consistent with the loading, sliding and unloading of the probes. When the indentation depth of the stimulus into the sensor surface is increased, the amplitude of the peak value obtained with short time Fourier analysis on the output signal also increases. This increase continues until the indentation depth is maintained constant when it is observed that the signal appears to relax. When the stimulus is retracted and immediately indented to the previous depth, the signal decreases rapidly and then returns to its former value at a rate consistent with the stimulus speed. The data for the control sensor (alginate without fibroblasts) show a lower grade of coherence with the test protocol, therefore suggesting that the integration of the fibroblasts cellular elements enhanced the effectiveness of the mechanotransduction process.

The protocol was implemented under position control along the indentation direction. This is because the mechanical properties of alginate hydrogels are complex, poorly understood and very transient, and the feedback needed for force controlled experiments could result in unstable indentation depth profile and difficult to analyse data, particularly in combination with a tangential sliding motion. Each channel reacted independently, so in principle the data are indicative of the local environment. However, the device is comprised of a 1D impedance sensor array covered with a 3 mm thick layer of artificial tissue and with a further 3 mm thick layer of PDMS. When a stimulus is brought into contact with the PDMS layer so that the displacement of the centre of the layer is 1 mm, the PDMS is indented but also bends like a plate on a soft substrate. This means that the size of the resulting sub-surface stress field will be much greater and more uniform than that which would be expected for a similar indentation into a PDMS half-space. If the impedance is proportional to the mean hydrostatic stress within the tissue, the relative uniformity of the stress field may explain why the changes in impedance seem to be comparable over all channels. Essentially, the phenomenon may be regarded as a direct consequence of Saint-Venant's principle. The spatial sensitivity could be improved by reducing the thickness of the PDMS layer, and hence increasing the stress gradients in the stress field, through enhanced fabrication methods.

In conclusion, the preliminary results presented in this work deserve further evaluation while clearly showing that the system is appropriate to conduct the targeted experiments, since the output plots track the expected behaviour related to the protocol. These achievements in assessing the feasibility of this novel system, that is one of the first bio-hybrid tactile transducers presented in the literature, can be considered as a preliminary step towards the development of a bio-hybrid sensor for prosthetic applications [49], which combines biological and artificial materials [50]. At the moment, the system does not implement automatically controlled homeostasis regulation, however this is a planned action that will be implemented in future devices: as a matter of fact, the device actually has the ability to supply fresh medium and remove waste products through its microfluidic system as well as space to incorporate a Peltier plate to maintain the temperature. Further work will also examine a range of cells and stimuli and the current device could be also used as a platform for such studies and also to enhance the scientific knowledge of the behaviour of skin analogues with encapsulated cells.

## Figures and Tables

**Table 1.** Comparison between different approaches towards artificial tactile sensing, adapted from [13].

**ARTIFICIAL TACTILE SENSING**			
**Approaches**	**Types**	**Advantages**	**Disadvantages**
***FULLY-SYNTHETIC tactile sensing***	Capacitive sensors Piezo electric sensors Piezoresistive sensors Inductive sensors Optoelectric sensors Strain gauge sensors	Physical robustness Greater sensitivity Simple integration	Non self-healing properties Biocompatibility assessment
***BIO-HYBRID tactile sensing***	Silicon-based bio-hybrid sensor with microfluidics and conductivity sensors Silicon-based MEMS sensors with skin analogue Polymeric substrate with bio-hybrid skin-like electrode	Bio-mimicry Bio-inspiration Self-healing Greater softness and compliance	Conservation of living cells Biocompatibility assessment
***FULLY-BIOLOGICAL tactile sensing***	Skin analogue including mechanoreceptor cells directly interfaced to the residual nervous system of the amputee	Bio-mimicry Bio-inspiration Self-healing Wettability Regeneration of tissues Greater softness and compliance Biocompatibility Biodegradability	Conservation of living cells Rejection Complex integration Variability

**Table 2:** Etch and passivation parameters used in the fabrication of the microfluidic channels.

	**Etch Cycle**	**Passivate Cycle**
SF_6_ Flow Rate	100 sccm	0 sccm
C_4_F_8_ Flow Rate	0 sccm	85 sccm
Duration	12 s	5 s
13.56 MHz Platen Power	800 W	600 W
RF Coil Power	600 W	600 W

**Table 3.** Coefficient of determination for control sensor (*i.e.*, alginate only).

**Test**	**Channel**										**Average**

	**1**	**2**	**3**	**4**	**5**	**6**	**7**	**8**	**9**	**10**	
A	0.240	0.423	0.325	0.726	0.681	0.682	0.701	0.663	0.681	0.246	0.537
B	0.548	0.697	0.307	0.612	0.537	0.429	0.629	0.711	0.715	0.618	0.580
C	0.444	0.287	0.299	0.510	0.546	0.425	0.512	0.662	0.674	0.430	0.479

**Table 4.** Coefficient of determination for test sensor (*i.e.*, alginate with fibroblasts).

**Test**	**Channel**										**Average**

	**1**	**2**	**3**	**4**	**5**	**6**	**7**	**8**	**9**	**10**	
D	0.985	0.646	0.983	0.843	0.834	0.923	0.935	0.936	0.758	0.984	0.882
E	0.983	0.758	0.863	0.861	0.839	0.819	0.820	0.841	0.899	0.985	0.862
F	0.983	0.936	0.862	0.875	0.652	0.781	0.781	0.887	0.907	0.983	0.865
